# Body Mass Index and Prevalence of Obesity in Brazilian Adult Women: Temporal Comparison of Repeated Population-Based Cross-Sectional Surveys

**DOI:** 10.1155/2024/9950895

**Published:** 2024-10-29

**Authors:** Anderson Garcez, Juvenal Soares Dias-da-Costa, Fernanda Souza de Bairros, Maria Teresa Anselmo Olinto

**Affiliations:** ^1^Post-Graduate Program in Nutrition Sciences, Federal University of Health Sciences of Porto Alegre, UFCSPA, Porto Alegre, Rio Grande do Sul, Brazil; ^2^Post-Graduate Program in Collective Health, University of Vale do Rio dos Sinos, UNISINOS, São Leopoldo, Rio Grande do Sul, Brazil; ^3^Department of Collective Health, Federal University of Rio Grande do Sul, UFRGS, Porto Alegre, Rio Grande do Sul, Brazil; ^4^Post-Graduate Program in Food, Nutrition and Health, Federal University of Rio Grande do Sul, UFRGS, Porto Alegre, Rio Grande do Sul, Brazil; ^5^Post-Graduate Program in Medical Sciences: Endocrinology, Federal University of Rio Grande do Sul State, UFRGS, Porto Alegre, Rio Grande do Sul, Brazil

**Keywords:** body mass index, body weight, obesity, prevalence, women's health

## Abstract

**Background:** Obesity is a complex multifactorial disease that has been associated with higher morbidity and mortality.

**Objectives:** This study aimed to compare changes in body mass index (BMI) and obesity prevalence between two cross-sectional samples of Brazilian women. Furthermore, retrospective assessments of lifetime body weight changes were explored.

**Methods:** Two independent population-based cross-sectional surveys were conducted in 2003 (first survey) and 2015 (second survey) with women living in the urban area city in southern Brazil. Both surveys had a similar design and included 981 women aged 20–60 years. Mean BMI and the presence of obesity (BMI ≥ 30 kg/m^2^) were estimated. Additionally, lifetime body weight change was obtained for the retrospective longitudinal assessment.

**Results:** After 12 years, there was a significant increase from 25.9 ± 5.3 kg/m^2^ to 28.1 ± 6.2 kg/m^2^ in mean BMI. Between 2003 and 2015, the prevalence of obesity increased by 73% (18.0%; 95% CI: 15.8–20.6 vs. 31.2%; 95% CI: 28.3–34.1; *p* < 0.001). The means of estimated cumulative body weight gain from 15 to 50 years were 15.2 kg (95% CI: 13.3–17.1) and 17.2 kg (95% CI: 15.5–18.9) in 2003 and 2015, respectively; the greater cumulative difference between the two periods was observed at 40 years of age (3.3 kg).

**Conclusions:** There was a significant increase in the mean BMI and prevalence of obesity between 2003 and 2015. Moreover, women experienced higher body weight gain during their lives in both survey periods, mainly in early adulthood.

## 1. Introduction

Obesity is a complex multifactorial disease that has been increasing worldwide in the last few decades [1–3]. In addition, excess body weight (BW) and weight gain throughout life have been associated with higher morbidity and mortality [4–7].

The worldwide prevalence rates of overweight and obesity have approximately doubled since 1980 to an extent that over one-third of the world's population is now classified as overweight or obesity [3, 8]. Globally, the proportion of individuals with overweight increased between 1980 and 2015 from 25.4% to 38.5% in men and from 27.8% to 39.4% in women. The prevalence of obesity, from 1980 to 2015, increased from 5% to 10.1% in men and from 8.9% to 14.8% in women [3]. The prevalence rates of overweight and obesity were always greater in women than in men throughout this period; a pattern of diminishing sex differences in recent years was evident for overweight, but sex differences in obesity remained remarkably constant [3].

In low-income countries, obesity mostly affects middle-aged adults (especially women) from wealthy, urban environments, whereas in high-income countries, it affects both sexes and all ages, but is disproportionately greater in disadvantaged groups [9]. The frequency of obesity is higher in women than in men in all sociodemographic levels [10]. Additionally, the prevalence of obesity has increased in Brazil, mainly among women [11, 12].

A substantial tracking of overweight and obesity from childhood into adulthood has been demonstrated [13–15]. The BW trend shows a tendency to increase during different stages of life, that is, BW increases during the transition to adulthood, and these increases grow larger over time, especially in women [16]. It is clear that hormonal fluctuations during the female life span may explain the increased risk of weight gain and obesity in women [17]. In addition, excessive weight gain throughout life has been associated with various sociodemographic factors such as race and socioeconomic status [16, 18].

In this study, we aimed to compare the temporal changes in body mass index (BMI) and obesity prevalence among adult Brazilian women from two independent cross-sectional surveys performed in 2003 and 2015, respectively. Furthermore, retrospective assessments of lifetime BW changes were explored. Our main research question is whether there will be differences in the prevalence of obesity and lifetime BW changes between the two surveys. We hypothesize that the prevalence of obesity will be higher in 2015 compared to 2003 and that greater BW gain will primarily occur during early adulthood. This study is justified by the growing public health concern surrounding obesity and the need for descriptive comparative data to guide effective surveillance, prevention, and intervention strategies, particularly among women.

## 2. Materials and Methods

Two repeated and independent population-based cross-sectional surveys with random representative samples of adult women living in the urban area of the municipality of São Leopoldo were conducted in 2003 (the first survey) and 2015 (the second survey). This municipality is located in the Rio dos Sinos Valley in southern Brazil, within the metropolitan area of Porto Alegre (the capital of Rio Grande do Sul State). Both surveys had a similar design and were used to compare temporal changes in BW, BMI, and prevalence of obesity.

### 2.1. Samples

The 2003 sample was carried out in multiple stages [19]. Initially, 40 census sectors were systematically selected from the 270 sectors in the municipality. In each conglomerate, blocks and corners were drawn to start the survey. The inclusion criteria for participating were all women aged 20–60 years living in the selected households. A sample size of adult women was calculated for a prevalence study of diabetes mellitus, which allowed the identification of a risk ratio of 2.0, with a confidence level of 95% and statistical power of 80%. To compensate for potential losses/refusals during fieldwork and for confounder control, the estimated required sample size was increased by 25%; thus, the sample totaled 1084 women.

The 2015 sample was carried out in multiple stages [20]. The 371 census tracts in the urban area of São Leopoldo were classified in descending order from the sector with the highest monthly nominal income of people aged 10 years and older (with or without income), according to the Brazilian Institute of Geography and Statistics (IBGE). A total of 45 census tracts were systematically selected. In each conglomerate, blocks and corners were drawn to start the survey. The inclusion criteria for participating were all women aged 20–69 years living in the selected households. The sample size was determined according to the outcome that required the highest number of participants (delayed cytopathological examination). It was calculated considering an odds ratio of 2.0, a confidence level of 95%, a statistical power of 80%, and a nonexposed/exposed ratio of 1:2. The calculated sample size was increased by 25%: 10% to compensate for losses and refusals and 15% to control for confounding factors leading to a sample total of 1281 women.

Both surveys excluded women who did not live within the drawn census sectors, pregnant women, and those with physical or mental disabilities to answer the questionnaire (exclusion criteria) [21]. Both surveys were conducted according to the guidelines laid down in the Declaration of Helsinki, and all procedures involving human subjects were approved by the Ethics and Research Committee of the University of Vale do Rio dos Sinos, UNISINOS (Certificate of Presentation for Ethical Appreciation number: 30872914.6.0000.5344). Written informed consent was obtained from all subjects. After excluding women aged 61–69 years from the second survey (2015) and those with anthropometric data loss, the final analysis of each study included 981 women aged 20–60 years [19, 20].

### 2.2. Data Collection

Data were collected throughout March and December 2003 (the first survey), and February and October 2015 (the second survey). In both the first and second surveys, data were collected via face-to-face interviews during home visits, using a standardized, precodified, and pretested questionnaire developed by the authors. The questionnaires included demographic, socioeconomic, reproductive, and anthropometric characteristics. All interviewers were trained and participated in a pilot study conducted in a nonselected census tract. Reliability was assessed by readministering the questionnaire to a random sample of 10% of the participants and was not subject to short-term changes. The collected data were entered twice using EpiData software and checked for typographical errors.

### 2.3. Outcome Variables

All outcome variables were obtained using the same methods in the 2003 and 2015 cross-sectional surveys. The outcome variables were the means of BMI and obesity prevalence (BMI ≥ 30 kg/m^2^). BMI (kg/m^2^) was obtained via direct anthropometric measurements of BW and height. BW was measured to the nearest 0.1 kg with the subject in light indoor clothes, with emptied pockets and without shoes, using a calibrated digital anthropometric scale (Omron model HN-289LA). Body height was measured without shoes to the nearest 0.5 cm using a portable stadiometer (Seca Bodimeter 208, Hamburg, Germany). BW and height were measured twice for each participant, and the average of the two values was used. Two variables were obtained from BMI: continuous BMI and dichotomous BMI (≥30 kg/m^2^; prevalence of obesity) [22, 23]. Continuous BMI variables and obesity prevalence were compared between the two cross-sectional surveys (temporal changes between 2003 and 2015). Furthermore, retrospective assessments of lifetime outcomes were BW change, cumulative BW gain, and lifetime BMI change. BW change was obtained by self-reported BW (kg) in a retrospective longitudinal assessment of lifetime BW change. Each woman was asked to report their BW at different stages of life (at age 15, 20, 30, 40, and 50 years), up until the current age at the time of each survey. The cumulative BW gain was obtained by the difference in BW between age 15 and the other stages of life (at age 20, 30, 40, and 50 years). In addition, lifetime mean BMI change was estimated using retrospective self-reported BWs and measured heights obtained at the time of survey application.

### 2.4. Explanatory Variables

Both surveys included demographic, socioeconomic, and reproductive variables to characterize the samples and to analyze the distribution of outcomes. The sociodemographic variables used in the two surveys were age (20–30, 31–40, 41–50, and 51–60 years); skin color (self-reported according to the categories proposed by the IBGE [white, black, brown, yellow, and indigenous] [24] and later grouped into “white” and “others” [black/brown/yellow/indigenous]); civil or marital status, which included with partner (married) and without partner (single, divorced, or widowed); year of study (schooling), which included high (≥ 11 years = secondary or higher), medium (8–10 years = intermediate), and low (≤ 7 years = elementary incomplete); economic class, which included high (A/B class), middle (C class), and low (D/E class) based on the Brazilian Association of Research Companies (ABEP) scale that estimated the buying power of individuals and families, including the possession of household items and the education level of the household; per capita family income in minimum wages (> 3 minimum wages, 1.01–3 minimum wages, 0.51–1 minimum wages, and ≤ 0.5 minimum wages); and occupation (working and not working). Reproductive variables included the number of pregnancies (nulliparous, 1–2, 3–4, and ≥ 5), referring to the total number of pregnancies during the woman's reproductive life, including abortions, stillbirths, and live births) and age at menarche (8–11, 12–13, and ≥ 14 years).

### 2.5. Data Analyses

Descriptive statistics, including absolute and relative frequencies, mean, and standard deviation (SD), were used to describe and compare the sample's characteristics, prevalence of obesity, and mean BMIs between the two cross-sectional surveys (2003 and 2015). Pearson's chi-square test was conducted to compare prevalence rates, while Student's *t*-test was used to compare the mean BMIs. Bivariate regression analysis was also conducted. Linear regression was used with mean BMI as a dependent variable and sociodemographic and reproductive characteristics as independent variables. This regression estimates the magnitude of change, beta regression (*β*) with 95% confidence intervals (95% CI), in relation to a reference group for each factor independent of the other variables. Poisson regression with robust variance estimates was performed using the prevalence of obesity as a dependent binary variable sociodemographic and reproductive characteristics as independent variables. This regression presents the magnitude of change in relation to a reference group as a ratio (prevalence ratio [PR]) with 95% CI.

To explore the retrospective longitudinal lifetime BW and BMI changes, all variables were analyzed and estimated, respectively, in kg and kg/m^2^ mean scores. A one-way repeated-measures ANOVA test was conducted to determine whether there were differences between the two cross-sectional surveys (2003 and 2015) across the five stages of life (at age 15, 20, 30, 40, and 50 years). For the repeated measures, the following parameters were used: within-subject factor name “timepoint” with 5 levels (at age 15, 20, 30, 40, and 50 years) for the independent variable and measure name “BW” and “BMI” for the dependent variable. Pairwise comparisons were interpreted on the “timepoint” plot (horizontal axis defined factor “timepoint”). Finally, a post hoc test of trends was used to obtain a significant within-subject time effect.

All data analyses were performed using Stata (Version 15.0; StataCorp, College Station, TX, USA). Statistical significance was set at *p* < 0.05.

## 3. Results

The data of 981 women aged 20–60 years (38.5 ± 11.1 years) from the first survey (2003) and 981 women aged 20–60 years (40.4 ± 11.4 years) from the second survey (2015) were analyzed in this study. The general characteristics of the participants for the two surveys are presented in Table 1. There was an increase in the proportion of women in the older age category (over 50 years of age) and a reduction in the youngest age category (20–30 years old) in 2015 when compared to 2003. In addition, a higher proportion of women were observed between the categories of nonwhite skin color, high years of study, economy class C, low per capita family income, 1–2 number of pregnancies, and age at menarche (8–11 years old) (Table 1).

After 12 years, there was a significant increase from 25.9 ± 5.3 kg/m^2^ to 28.1 ± 6.2 kg/m^2^ in mean BMI, whereas the prevalence of obesity increased 73% from 2003 (18.0%; 95% CI: 15.8–20.6) to 2015 (31.2%; 95% CI: 28.3–34.1; *p* < 0.001). Table 1 shows the comparison of mean BMIs and the regression analysis results according to sociodemographic and reproductive variable categories between 2003 and 2015. Mean BMIs increased significantly across all categories of variables (except for pregnancies numbering five or more). In 2003, mean BMIs less than or equal to 25 kg/m^2^ (eutrophic) were observed in some categories, whereas in 2015, all categories showed mean BMIs over 25 kg/m^2^.  Table 2 shows that obesity prevalence doubled among younger women who did not live with a partner, with high education, higher economic class, lower family income, among those who work, and those with 3–4 pregnancies when comparing both surveys.

A higher mean BMI and prevalence of obesity were observed, especially among those women who were older, had lower schooling, lower family income, with higher number of pregnancies, and early menarche in both survey periods, whereas all other variables did not reveal a statistical consistency between the two surveys (Tables 1 and 2). In the first survey, BMI and obesity were most commonly associated with age, schooling, economic class, family income, and occupation; in the second survey, only age and schooling associations with BMI and obesity remained. Reductions in the magnitude of the associations were particularly evident in the second survey (2015).

**Figure 1:**
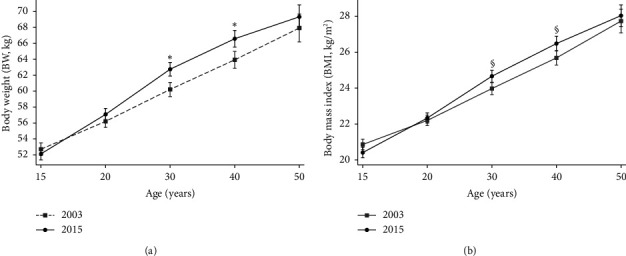
Mean and 95%CI for BW (Graph (a)) and BMI (Graph (b)) from age 15 and 50 years by the two survey periods (2003 and 2015). The results showed a statistically significant increasing linear trend—time effect—for BW and BMI in both survey periods (*p* < 0.001 for one-way repeated-measures ANOVA test). ⁣^∗^*p* < 0.001; ^§^*p* < 0.01 for differences between the two cross-sectional surveys (2003 and 2015).

The retrospective longitudinal measures of BW changes are shown in Table 3. Except at age 15, all other age groups had higher mean BW in women in the 2015 survey than in 2003. In 2003, from 15 to 30 years of age, there was an estimated weight gain of 7.5 kg, whereas in 2015 for the same age period, the weight gain was 10.7 kg. The means of estimated cumulative BW gain from 15 to 50 years were 15.2 kg (95% CI: 13.3–17.1) and 17.2 kg (95% CI: 15.5–18.9) in 2003 and 2015, respectively; the greater cumulative difference between the two periods was observed at 40 years of age (3.3 kg) (Table 3). A one-way repeated-measures ANOVA test revealed a statistically significant increasing linear trend—time effect—for weight and BMI in both survey periods (Figure 1). A significant difference in mean BW and BMI between the two periods was observed at 30 and 40 years of age. The mean BMI was higher than 25 kg/m^2^ between 30 and 40 years of age.

## 4. Discussion

The purpose of this study was to assess temporal changes in BMI and obesity prevalence in Brazilian adult women from two independent cross-sectional surveys conducted in 2003 and 2015, respectively. Each of the two studies comparing these changes showed a high proportion of women with obesity. Over the 12-year period, there was a significant increase in the mean BMI, whereas the prevalence of obesity increased by 73%. In addition, significant changes in BW and BMI were observed across the phases of life.

The present study showed an increase in the prevalence of obesity in Brazilian adult women, consistent with other studies conducted in developing countries [25] as well as in Brazil [11, 12]. The prevalence of overweight and obesity increased in both sexes from 2002 to 2013, based on data from three Brazilian nationwide surveys (the Household Budget Survey 2002/2003 and 2008/2009, and the National Health Survey 2013) [12]. The prevalence of obesity increased from 7.5% to 17.0% from 2002 to 2013 among adults aged 20–39 years and from 14.7% to 25.7% among those aged 40–59 years. Women had a higher prevalence of obesity compared to men in all surveys [12]. Additionally, data from two population-based surveys conducted in 1995 and 2005 in the city of Rio de Janeiro, Brazil, showed a sharp increase in obesity in the 10-year period (16.6%–24.0%) among women aged 35 years or older [11]. Reasons for this increase of obesity are complex and relate to environmental factors, such as diet and physical activity, for example. Studies have reported an overall increase in energy intake and a reduction in energy expenditure over time among the population [1, 25–27].

In this study, we conducted bivariate analyses with sociodemographic and reproductive factors; however, previous studies have explored these relationships more extensively [19, 20]. Notably, elevated BMI and obesity were most commonly negatively associated with education, economic class, and occupation in the first survey (2003), whereas decreases in BMI and obesity measure rates were observed in the second survey. In developed countries, obesity among women was most commonly negatively associated with education and occupation, while positive associations for women in developing countries were most common with income and material possessions [28, 29]. Although the burden of obesity in developing countries tends to shift toward the groups with lower socioeconomic status as the country's gross national product increases, the shift of obesity toward women with low socioeconomic status apparently occurs at an earlier stage of economic development than it does for men [29]. Additionally, a consistent inverse relationship between life course socioeconomic status and obesity has been observed among women, but not among men [30]. Regarding reproductive factors, both a higher number of pregnancies and early menarche have been shown to be associated with elevated BMI and obesity. These associations were previously reported, indicating that these reproductive characteristics may play a significant role in influencing long-term BW regulation and obesity risk [31, 32].

A higher mean BMI and prevalence of obesity were observed, especially among older women. In addition, the retrospective longitudinal lifetime BW and BMI changes showed a significant increase across phases of life; there was an estimated weight gain of 7.5 kg from 15 to 30 years of age in 2003, and weight gain of 10.7 kg in 2015. The mean BMI was also observed to be higher than 25 kg/m^2^ between 30 and 40 years of age. In summary, our results showed that BMI and the prevalence of obesity increased with age. In this sense, the rates of both overweight and obesity generally increased starting with the age of 20 years onward, reached their peak between the ages of 50–65 years, and declined slightly thereafter [3]. Additionally, a substantial tracking of overweight and obesity from childhood into adulthood has been demonstrated in several studies [13–15]. The BW trend shows tendency to increase along with the stages of life, i.e., BW increases during the transition to adulthood and these increases grow larger over time, especially in women [16]. The prevalence of overweight was somewhat lower in women than in men among young adults (aged between 20 and 44 years), but this trend was reversed after age 45–49 years, perhaps coinciding with menopause in women [3]. Weight gain is common among aging women, especially during the menopausal transition; aging results in a decrease in lean body mass, which decreases the resting metabolic rate [33]. Hormonal fluctuations during the female life span may explain the increased risk for weight gain and obesity in women [17]. It is also likely that dietary intake changes at menopause and that women who are particularly at risk for gaining weight at this time may exhibit decreased energy expenditure in combination with increased total food intake because reproductive hormones influence eating behavior and food preferences [17].

In this study, we used probabilistic and representative samples of adult Brazilian women from two independent population-based cross-sectional surveys. In addition, both surveys were carried out using a similar design and methodology to ensure correctness and uniformity in the information collection and to make the surveys comparable. Despite these strengths, the results of this investigation need to be interpreted within the context of some limitations. First, this study was conducted on a population of adult women; thus, our findings may not be generalizable to other population groups. Second, current BW and height were measured, and BW at 15, 20, 30, 40, and 50 years was assessed retrospectively (every woman self-reported their BWs according to the current age at the time of application of the surveys). Thus, the retrospective nature of the data introduces certain limitations, particularly regarding the accuracy of individual-level BMI estimates [34]. Additionally, retrospective assessments of lifetime BW may be influenced by recall errors over longer periods, as the ability to recall earlier experiences tends to diminish with age. Third, the retrospective longitudinal lifetime BMI change was estimated considering self-reported BWs and measured heights obtained at the time of application of the surveys; that is, we consider the current height for estimating BMI retrospectively. If so, this would lead to misclassification. However, women did not show significant changes in height after 15 years of age; a dramatic reduction in bone mass growth is observed after this age, particularly in women [35, 36]. Fourth, another limitation is that, while BMI is generally a good proxy for estimating body fatness at the group level, significant variations exist between groups depending on socioeconomic status, educational attainment, physical activity patterns, and age [37, 38]. Fifth, owing to the cross-sectional design, the associations of sociodemographic and reproductive characteristics with BMI and obesity should be interpreted with caution. Finally, this investigation did not aim to assess dietary habits or physical activity throughout life, which could have an impact on weight gain; we only sought to compare a temporal trend in terms of anthropometric data.

## 5. Conclusions

In this study, we revealed a high proportion of women with obesity, as well as a temporal increase in BMI and prevalence of obesity in Brazilian adult women, which were noted between 2003 and 2015. Over the 12-year period, there was a significant increase in the mean BMI, whereas the prevalence of obesity increased by 73%. In addition, significant BW and BMI increases were noted across phases of life, including early adulthood (from 15 to 30 years, for example). The cumulative BW gains from 15 to 50 years were estimated to be 15.2 kg and 17.2 kg in the surveys of 2003 and 2015, respectively. These findings indicate that adult women have a higher prevalence of obesity, such as lifetime weight gain. Future studies should be planned to provide a detailed description of the changes in BW and BMI, particularly among female populations. Additionally, this study reinforces obesity in early female adulthood as a relevant topic in the public health agenda in Brazil.

## Figures and Tables

**Table 1:** Characteristics of the samples, BMI mean ± standard deviation (SD), and regression analysis results for mean BMI according to demographic, socioeconomic, and reproductive characteristics, stratified by survey periods (2003 and 2015), among women from São Leopoldo, southern Brazil.

Characteristics			2003		2015	
	*n* (%)		BMI (kg/m^2^)		BMI (kg/m^2^)	
	2003	2015	Mean ± SD	*β* (95% CI)⁣^∗∗∗^	Mean ± SD	*β* (95% CI)⁣^∗∗∗^
Total sample	*N* = 981	*N* = 981	25.9±5.3		28.1±6.2⁣^∗∗∗^	
Age (years)				*< 0.001*		*< 0.001*
20–30	286 (29.2)	236 (24.1)⁣^∗^	24.3 ± 4.4	1.00	25.9 ± 5.7⁣^∗∗^	1.00
31–40	252 (25.7)	256 (26.1)	25.5 ± 5.1	1.26 (0.39, 2.13)	28.3 ± 6.3⁣^∗∗^	2.42 (1.34, 3.50)
41–50	281 (28.6)	266 (27.1)	26.5 ± 5.5	2.22 (1.37, 3.06)	29.2 ± 6.4⁣^∗∗^	3.31 (2.24, 4.38)
51–60	162 (16.5)	223 (22.7)⁣^∗∗^	28.4 ± 5.7	4.16 (3.17, 5.15)	29.1 ± 5.9⁣^∗^	3.22 (2.10, 4.33)
Skin color⁣^∗∗∗∗^				*0.177*		*0.355*
White	825 (84.1)	727 (74.1)⁣^∗∗^	25.8 ± 5.1	1.00	28.0 ± 6.1⁣^∗∗^	1.00
Others	156 (15.9)	254 (25.9)⁣^∗∗^	26.4 ± 6.3	0.63 (−0.28, 1.54)	28.4 ± 6.7⁣^∗∗^	0.42 (−0.47, 1.31)
Marital status				*< 0.001*		*0.079*
Without partner	354 (36.1)	347 (35.4)	24.8 ± 5.0	1.00	27.7 ± 6.5⁣^∗∗^	1.00
With partner	627 (63.9)	634 (64.6)	26.5 ± 5.4	1.75 (1.06, 2.43)	28.4 ± 6.0⁣^∗∗^	0.73 (−0.08, 1.55)
Schooling (years of study)				*< 0.001*		*< 0.001*
≥ 11 (secondary or higher)	383 (39.0)	437 (44.6)⁣^∗^	24.6 ± 4.5	1.00	27.2 ± 5.8⁣^∗∗^	1.00
8–10 (intermediate)	160 (16.3)	181 (18.5)	25.4 ± 4.7	0.73 (−0.23, 1.69)	28.0 ± 5.9⁣^∗∗^	0.80 (−0.26, 1.87)
≤ 7 (elementary incomplete)	438 (44.7)	363 (37.0)⁣^∗∗^	27.2 ± 5.8	2.59 (1.87, 3.30)	29.3 ± 6.6⁣^∗∗^	2.16 (1.30, 3.00)
Economic class (ABEP scale)				*0.007*		*0.050*
A/B (high)	338 (34.4)	351 (35.8)	25.4 ± 4.7	1.00	27.3 ± 6.0⁣^∗∗^	1.00
C (middle)	387 (39.5)	511 (52.1)⁣^∗∗^	25.9 ± 5.3	0.52 (−0.26, 1.29)	28.7 ± 6.3⁣^∗∗^	1.34 (0.49, 2.18)
D/E (low)	256 (26.1)	119 (12.1)⁣^∗∗^	26.5 ± 6.0	1.19 (0.33, 2.05)	28.0 ± 6.3⁣^∗∗^	0.59 (−0.70, 1.88)
Family income (MW)	*n* = 969	*n* = 949		*0.003*		*0.001*
> 3	145 (15.0)	67 (7.1)⁣^∗∗^	24.8 ± 4.5	1.00	25.7 ± 5.5⁣^∗∗^	1.00
1.01–3	347 (35.8)	314 (33.1)	25.9 ± 4.9	1.08 (0.05, 2.11)	27.9 ± 5.8⁣^∗∗^	2.21 (0.57, 3.85)
0.51–1	286 (29.5)	337 (35.5)⁣^∗^	26.1 ± 5.1	1.34 (0.28, 2.49)	28.3 ± 6.4⁣^∗∗^	2.58 (0.95, 4.21)
≤ 0.5	191 (19.7)	231 (24.3)⁣^∗^	26.6 ± 6.7	1.79 (0.64, 2.93)	28.9 ± 6.7⁣^∗∗^	3.20 (1.51, 4.89)
Occupation				*< 0.001*		*0.063*
Working	565 (57.6)	599 (61.1)	25.3 ± 4.8	1.00	27.8 ± 5.9⁣^∗∗^	1.00
Not working	416 (42.4)	370 (38.9)	26.7 ± 5.9	1.36 (0.69, 2.03)	28.6 ± 6.7⁣^∗∗^	0.76 (−0.04, 1.56)
Number of pregnancies				*< 0.001*		*< 0.001*
Nulliparous	195 (19.9)	162 (16.5)	23.5 ± 4.3	1.00	25.1 ± 5.4⁣^∗^	1.00
1–2	420 (42.8)	468 (47.7)⁣^∗^	25.6 ± 4.8	2.06 (1.20, 2.93)	28.2	3.01 (1.93, 4.10)
3–4	279 (28.4)	258 (26.3)	27.1 ± 5.4	3.56 (2.63, 4.49)	29.8 ± 6.7⁣^∗∗^	4.63 (3.44, 5.82)
≥ 5	87 (8.9)	93 (9.5)	29.1 ± 6.7	5.61 (4.33, 6.90)	28.7 ± 5.6	3.56 (2.01, 5.10)
Age at menarche (years)		*n* = 973		*< 0.001*		*< 0.001*
≥ 14	347 (35.4)	291 (29.9)⁣^∗^	25.2 ± 5.1	1.00	26.9 ± 5.3⁣^∗∗^	1.00
12–13	442 (45.0)	445 (45.7)	25.8 ± 5.2	0.66 (−0.08, 1.40)	28.3 ± 6.4⁣^∗∗^	1.39 (0.48, 2.30)
8–11	192 (19.6)	237 (24.4)⁣^∗^	27.4 ± 5.7	2.26 (1.33, 3.19)	29.5 ± 6.7⁣^∗∗^	2.56 (1.50, 3.62)

Abbreviations: ABEP, Brazilian Association of Research Companies (Brazilian Economic Classification Criteria); BMI, body mass index; MW, per capita family income in regional minimum wages (2003 = BRL 312.00 and 2015 = BRL 1006.88).

⁣^∗^*p* < 0.05; ⁣^∗∗^*p* < 0.001 for chi-square test (comparison of proportions) or for Student's *t*-test (comparison of means) between the two survey periods (2003 and 2015).

⁣^∗∗∗^Beta-coefficient (*β*) value obtained by linear regression with the respective 95% confidence interval (95% CI) values.

⁣^∗∗∗∗^Self-reported according to the categories proposed by the Brazilian Institute of Geography and Statistic (IBGE) [white, black, brown, yellow, and indigenous] [23], and later grouped into “white” and “others” [black/brown/yellow/indigenous]).

**Table 2:** Prevalence and regression analysis results for obesity (BMI ≥ 30 kg/m^2^) according to demographic, socioeconomic, and reproductive characteristics, stratified by survey period (2003 and 2015), among women from São Leopoldo, southern Brazil.

Characteristics	2003		2015	
	BMI ≥ 30 kg/m^2^		BMI ≥ 30 kg/m^2^	
	%	PR (95% CI)⁣^∗^	%	PR (95% CI)⁣^∗^
Total sample	18.0		31.2⁣^∗∗^	
Age (years)		*< 0.001*		*< 0.001*
20–30	9.8	1.00	20.3⁣^∗∗^	1.00
31–40	13.9	1.42 (0.89, 2.26)	31.3⁣^∗∗^	1.54 (1.13, 2.10)
41–50	21.0	2.14 (1.41, 3.26)	35.7⁣^∗∗^	1.76 (1.30, 2.37)
51–60	34.0	3.47 (2.30, 5.24)	37.2	1.83 (1.35, 2.48)
Skin color⁣^∗∗∗^		*0.264*		*0.449*
White	17.5	1.00	30.5⁣^∗∗^	1.00
Others	21.2	1.21 (0.86, 1.70)	33.1⁣^∗∗^	1.08 (0.88, 1.33)
Marital status		*0.043*		*0.301*
Without partner	14.7	1.00	29.1⁣^∗∗^	1.00
With partner	19.9	1.36 (1.01, 1.83)	32.3⁣^∗∗^	1.11 (0.91, 1.36)
Schooling (years of school)		*< 0.001*		*< 0.001*
≥ 11 (secondary or higher)	11.2	1.00	24.9⁣^∗∗^	1.00
8–10 (intermediate)	18.1	1.61 (1.05, 2.49)	33.7⁣^∗∗^	1.35 (1.04, 1.75)
≤ 7 (elementary incomplete)	24.0	2.14 (1.54, 2.96)	37.5⁣^∗∗^	1.50 (1.22, 1.85)
Economic class (ABEP scale)		*0.003*		*0.117*
A/B (high)	13.9	1.00	26.8⁣^∗∗^	1.00
C (middle)	18.1	1.30 (0.93, 1.83)	34.4⁣^∗∗^	1.29 (1.04, 1.59)
D/E (low)	23.4	1.69 (1.19, 2.38)	30.3⁣^∗∗^	1.13 (0.82, 1.56)
Family income (MW)		*0.035*		*0.009*
> 3	12.4	1.00	13.4	1.00
1.01–3	17.9	1.44 (0.88, 2.34)	31.5⁣^∗∗^	2.35 (1.25, 4.41)
0.51–1	18.2	1.46 (0.89, 2.41)	31.5⁣^∗∗^	2.34 (1.25, 4.39)
≤ 0.5	22.0	1.77 (1.07, 2.95)	35.5⁣^∗∗^	2.64 (1.40, 4.97)
Occupation		*0.002*		*0.124*
Working	14.7	1.00	29.4⁣^∗∗^	1.00
Not working	22.6	1.54 (1.18, 2.01)	34.0⁣^∗∗^	1.16 (0.96, 1.40)
Number of pregnancies		*< 0.001*		*< 0.001*
Nulliparous	7.2	1.00	14.2⁣^∗∗^	1.00
1–2	16.4	2.29 (1.32, 3.96)	29.1⁣^∗∗^	2.04 (1.37, 3.07)
3–4	21.9	3.04 (1.75, 5.29)	43.4⁣^∗∗^	3.06 (2.04, 4.58)
≥ 5	37.9	5.28 (2.98, 9.36)	37.6	2.65 (1.67, 4.20)
Age at menarche (years)		*< 0.001*		*< 0.001*
≥ 14	13.3	1.00	23.7⁣^∗∗^	1.00
12–13	17.4	1.31 (0.94, 1.84)	31.7⁣^∗∗^	1.34 (1.04, 1.71)
8–11	28.1	2.12 (1.49, 3.02)	39.7⁣^∗∗^	1.67 (1.29, 2.17)

Abbreviations: ABEP, Brazilian Association of Research Companies (Brazilian Economic Classification Criteria); BMI, body mass index; 95%CI: 95% confidence interval; MW, per capita family income in regional minimum wages (2003 = BRL 312.00 and 2015 = BRL 1006.88).

⁣^∗^Prevalence ratio (PR) with the respective 95% confidence interval (95% CI) values obtained by Poisson regression with robust variance.

⁣^∗∗^*p* < 0.001 for chi-squared test (comparison of proportions) between the two survey periods (2003 and 2015).

⁣^∗∗∗∗^Self-reported according to the categories proposed by the Brazilian Institute of Geography and Statistic (IBGE) [white, black, brown, yellow, and indigenous] [23], and later grouped into “white” and “others” [black/brown/yellow/indigenous]).

**Table 3:** Estimated retrospective lifetime body weight (BW) change and cumulative BW gain from age 15, stratified by survey periods (2003 and 2015), among women from São Leopoldo, southern Brazil.

Survey period	15 years	20 years	30 years	40 years	50 years	*p* trenda
2003						
BW						
*N*	805	848	625	434	163	
Mean ± SD	52.7 ± 9.5	56.2 ± 9.5	60.2 ± 11.2	63.9 ± 13.1	67.9 ± 13.7	*< 0.001*
BW change						
*N*		759	575	388	149	
Mean (95% CI)		3.5 (2.4–4.6)	4.0 (2.8–5.2)	3.7 (2.3–5.1)	4.0 (1.9–6.0)	*< 0.001*
Cumulative BW gainb						
*N*	—	759	531	352	119	
Mean (95% CI)	—	3.5 (2.4–4.6)	7.5 (6.3–8.7)	11.2 (9.9–12.5)	15.2 (13.3–17.1)	*< 0.001*
*p* valuec	—	*< 0.001*	*< 0.001*	*< 0.001*	*< 0.001*	
**2015**						
BW						
*N*	838	863	662	450	219	
Mean ± SD	52.1 ± 9.4	57.1 ± 11.2	62.8 ± 12.8	66.6 ± 14.1	69.3 ± 14.8	*< 0.001*
BW Change						
*N*		803	620	417	197	
Mean (95% CI)		5.0 (3.9–6.0)	5.7 (4.5–6.8)	3.8 (2.5–5.2)	2.7 (0.9–4.6)	*< 0.001*
Cumulative BW gainb						
N	—	803	586	391	187	
Mean (95%CI)	—	5.0 (3.9–6.0)	10.6 (9.5–11.8)	14.5 (13.2–15.7)	17.2 (15.5–18.9)	*< 0.001*
*p*valuec	—	*< 0.001*	*< 0.001*	*< 0.001*	*< 0.001*	

*Note: N*, the number of women differs from one age group to another because every woman was asked to report their body weight in different stages of life (at ages 15, 20, 30, 40, and 50 years), according to the current age at the time of carried out each survey.

^a^ *p* values for linear trend obtained by one-way repeated-measures ANOVA test (time effect).

^b^Cumulative body weight gain from 15 years old by ANOVA test.

^c^ *p* values for mean difference obtained by one-way repeated-measures ANOVA.

## Data Availability

The data that support the findings of this study are available from the corresponding author upon reasonable request.
